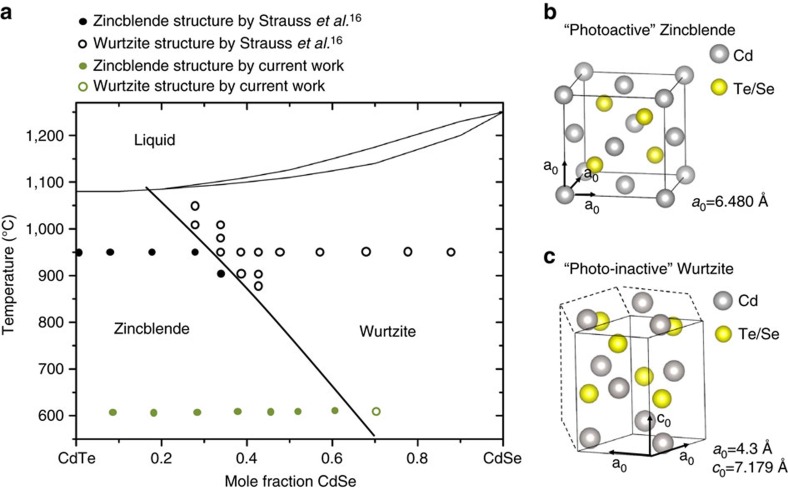# Corrigendum: Structural and compositional dependence of the CdTe_*x*_Se_1−*x*_ alloy layer photoactivity in CdTe-based solar cells

**DOI:** 10.1038/ncomms12831

**Published:** 2016-09-02

**Authors:** Jonathan D. Poplawsky, Wei Guo, Naba Paudel, Amy Ng, Karren More, Donovan Leonard, Yanfa Yan

Nature Communications 7: Article number: 1253710.1038/ncomms12537 (2016); Published: 07 27 2016; Updated: 09 02 2016

In Fig. 6 of this article, the division between the zincblende and wurtzite phases is misplaced in the low temperature range. The correct version of Fig. 6 appears below as Fig. 1.

## Figures and Tables

**Figure 1:**