# Supplementary material for: Structural and compositional dependence of the CdTexSe1−x alloy layer photoactivity in CdTe-based solar cells
Source: Nat Commun. 2016 Jul 27;7:12537. doi: 10.1038/ncomms12537 (PMC4974465; doi:10.1038/ncomms12537)
Supplement: Supplementary Information — Supplementary Figures 1-4 and Supplementary Tables 1-2 [file ncomms12537-s1.pdf]

Supplementary Figures

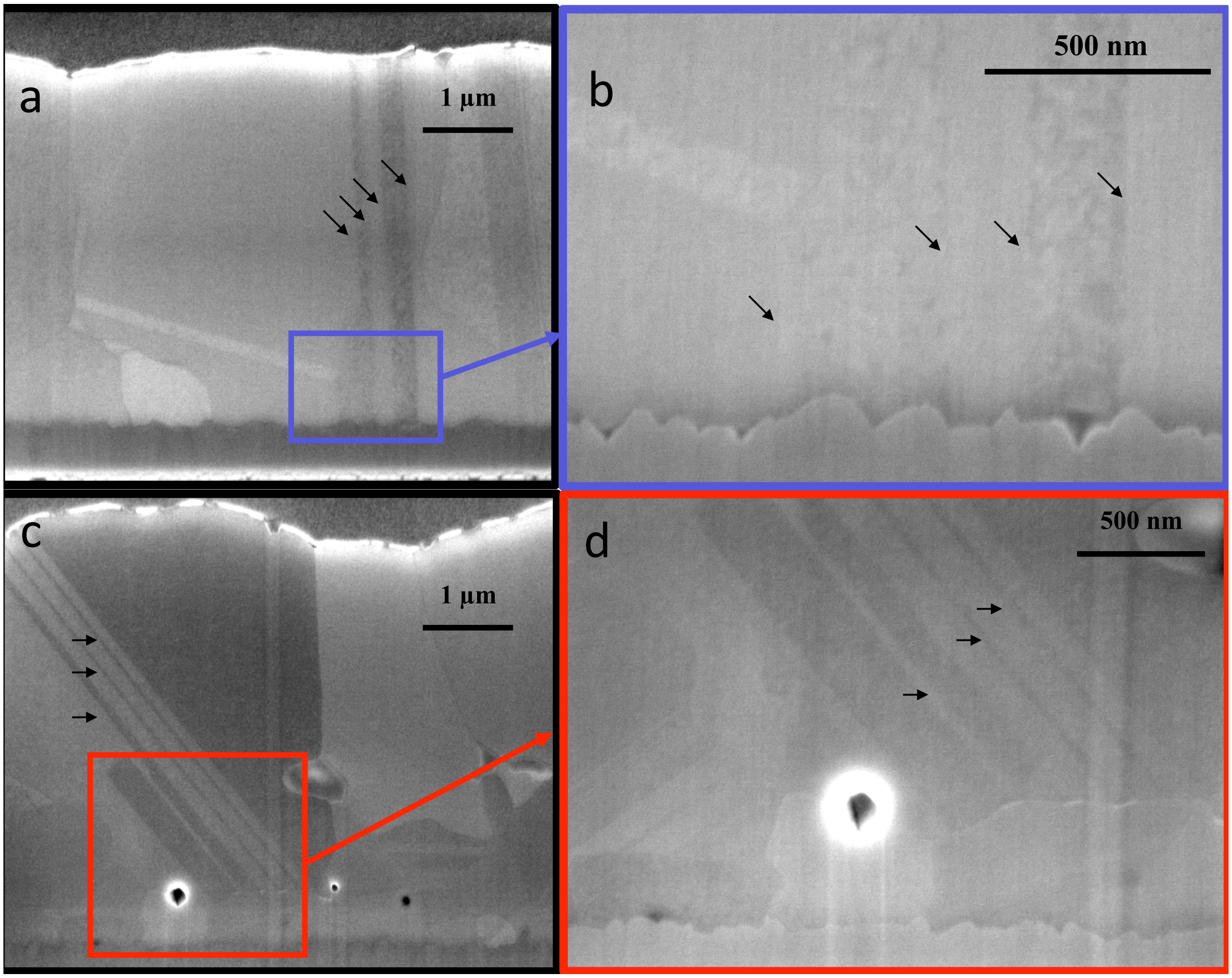

Supplementary Figure 1 | Cross-sectional Secondary Electron SEM Images: Cross-sectional SE-SEM images for the 100 nm (a,b) and 400 nm (c,d) CdSe layer samples. The columnar twin boundaries within a single CdTe grain marked with arrows directly interface the thin conducting oxide (FTO) layer. The SE-SEM image of this solar cell is similar to a SE-SEM image of a CdTe solar cell without a buffer layer, in which the CdTe grains directly interface the FTO layer. The 400 nm CdSe layer solar cell SE-SEM image also includes arrows pointing to several twin boundaries within a CdTe grain. These boundaries are terminated several 100 nm from the $CdTe_xSe_{1-x}$ /FTO interface on smaller polycrystalline grains. Data shown in Figure 4 of the main text confirms these grains as having the CdSe wurtzite structure. The large columnar grains are the CdTe-like zincblende structure.

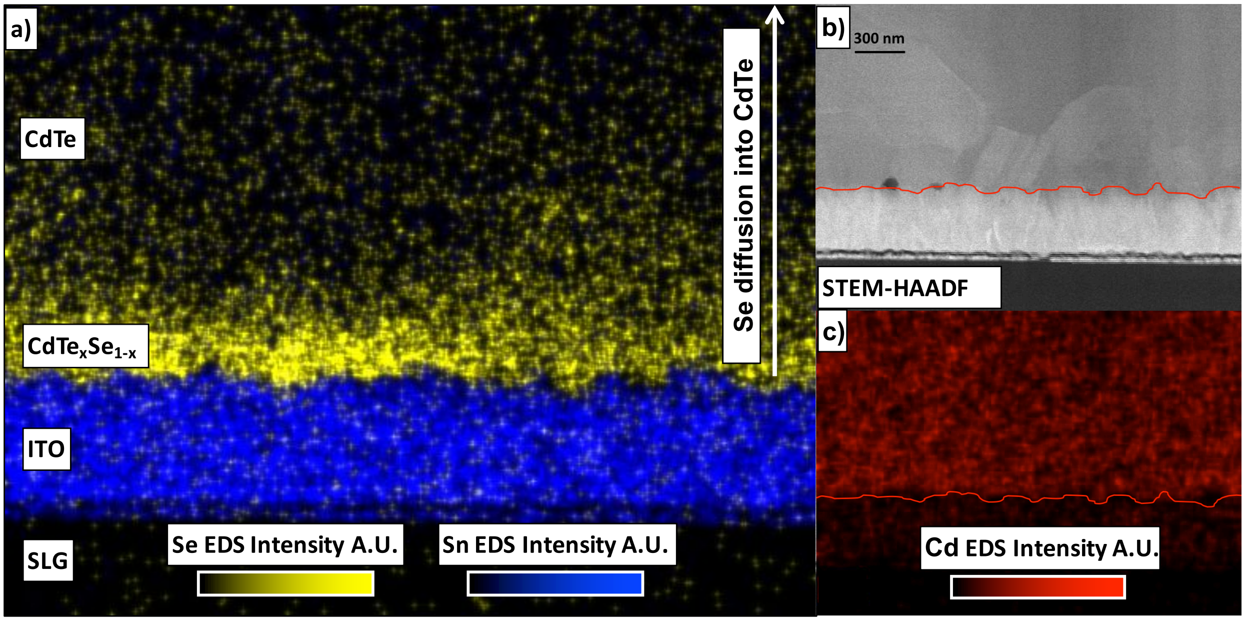

Supplementary Figure 2 | STEM-EDS of CdSe/CeTe Interface. a-c, (a) Se (yellow) and Sn (blue) STEM-EDS elemental intensity maps showing Se diffusion away from the FTO layer. (b) high angle annular dark field (HAADF)-STEM image with a simultaneously acquired (c) Cd EDS map where solid red lines designate the FTO/$CdTe_xSe_{1-x}$ interface for the 100 nm CdSe layer solar cell. Small Se-rich and Se-poor regions are observed within the $CdTe_xSe_{1-x}$ layer close to the FTO interface. As expected, the Cd map shows a homogenous distribution of Cd within the $CdTe_xSe_{1-x}$ layer. The Se-rich regions are consistent with APT measurements. The main advantage of STEM-EDS is the large field of view captured over one experiment (2x2 µm for this experiment), while APT are acquired from a much smaller volume (a 0.35 µm tall by 0.05 µm wide cylindrical volume for Figure 5 in the main text). However, APT is a more sensitive compositional technique and can better quantify the Se and Te compositional variations within small volumes compared to STEM-EDS.

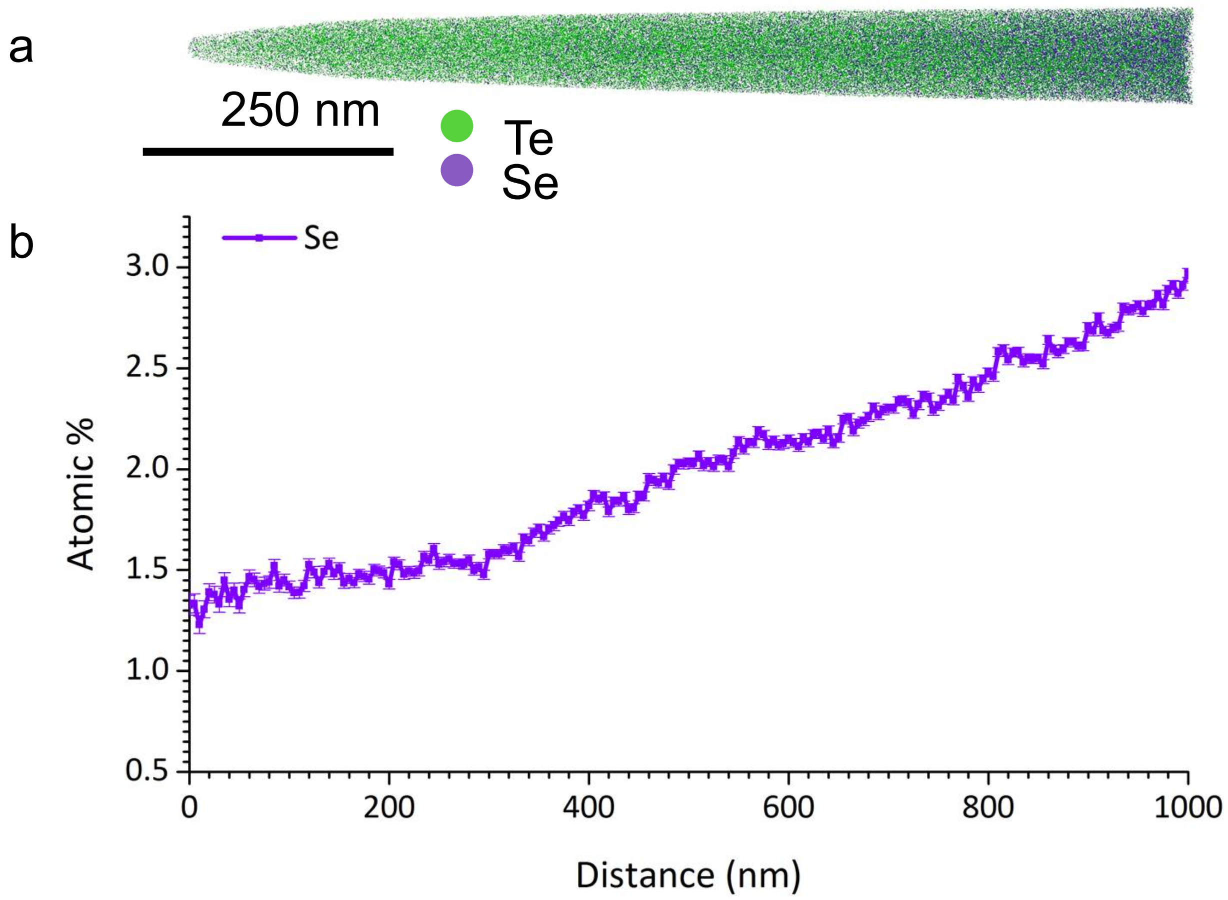

Supplementary Figure 3 | APT Showing the Extent of Se Diffusion. a-b, (a) APT reconstruction of a 1μm needle spanning from 1.3 μm – 2.3 μm from the back contact. The Se composition decreases with respect to distance in the CdTe layer in the growth direction. The distance marked 0 nm is 1.3 μm from the back contact, while 1,000 nm is approximately 2.3 μm from the back contact. The Se compositional data shown in Figure 5 of the main text only shows a small portion of the $CdTe_xSe_{1-x}$ region close to the FTO interface. The Se composition is greater than 10% at the lowest composition, and the line profiles indicate that the Se composition continues to decrease away from the FTO interface. Two additional APT needles were prepared at different depths from the back contact of the 100 nm CdSe layer sample to understand the Se composition with respect to distance from the interface. The CdTe thickness of this solar cell was 4 μm, and therefore the line profile extends from 2.7 μm to 1.7 μm from the FTO/$CdTe_xSe_{1-x}$ interface. The Se clearly diffuses into the CdTe layer several μms from the originally deposited CdSe layer. The error bars embedded in the 1D line profiles and proximity histograms are the standard error *(s)* from counting statistics, $s = \sqrt{\frac{c(1-c)}{N}}$, where *c* is the concentration of the element and *N* is the total number of ions.

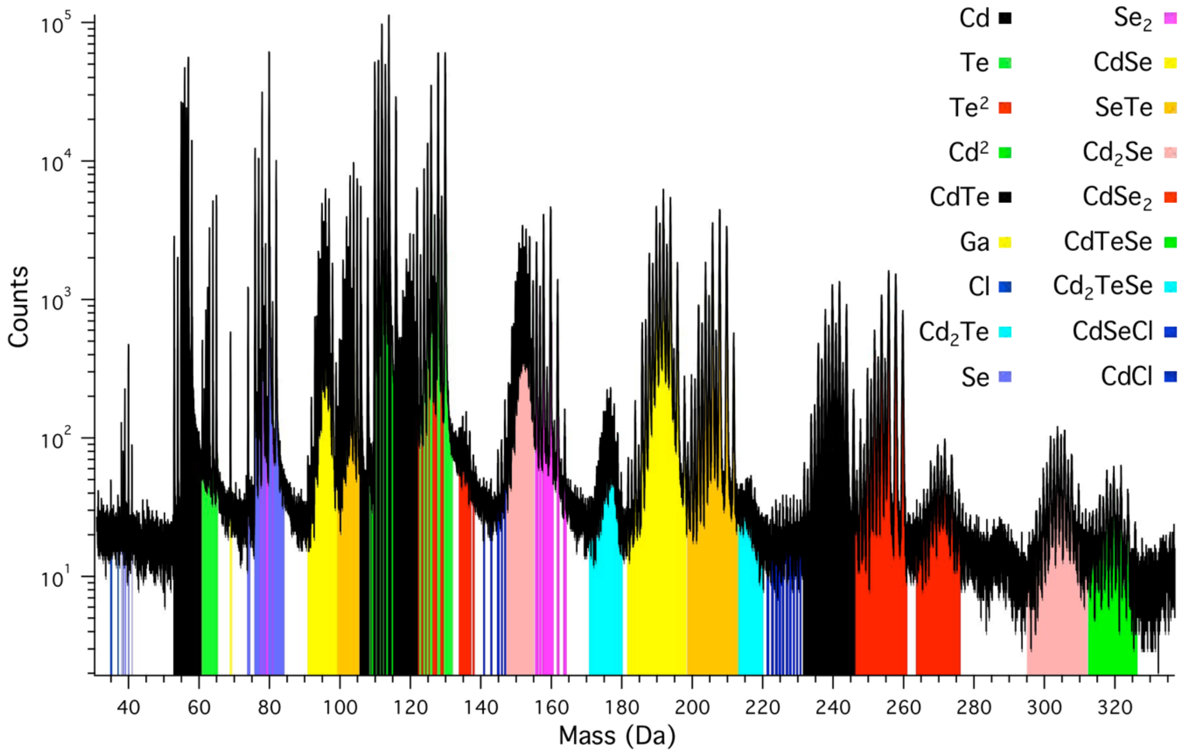

Supplementary Figure 4| The Mass Spectrum of CdTe/CdSe: The mass spectrum and associated ranges for the CdSe/CdTe solar cell APT dataset displayed in Figure 5 of the manuscript. The following ions and their charge states were ranged: Cd^{+}, Cd^{2+}, Te^{+}, Te^{2+}, Se^{+}, Se^{2+}, Te_2^{+}, Te_2^{2+}, Cd_2^{2+}, Se_2^{+}, Se_2^{2+}, $CdTe^{+}$, $CdTe^{2+}$, $CdSe^{+}$, $CdSe^{2+}$, $SeTe^{+}$, $SeTe^{2+}$, Cd_2Te^{2+}, Cd_2Se^{+} , Cd_2Se^{2+}, $CdSe^{2+}$, $CdSe_2^{2+}$, $CdTeSe^{+}$, Cd_2TeSe^{2+}, Ga^{+} (to assess FIB damage), Cl^{+}, $CdCl^{+}$, and $CdSeCl^{+}$. Several minor peak overlaps exist, but the major peaks accounting for most of the counted atoms do not have significant peak overlaps, and therefore, the minor peak overlaps for the major elements (Cd, Se, and Te) were ignored in the 1D line profiles and proximity histograms. For example, Te^{+} and Te^{2+} overlap, but there is significantly more Te^{+} counts compared to the Te^{2+}, and this overlap was ignored and ranged as Te^{+} for the 1D line profiles and proximity histograms. The overlaps that were manually decomposed for the 1D line profiles are the Cd_2Se^{2+}/CdCl and the CdTe/CdSeCl due to the low abundance of Cl.

Supplementary Tables

Ion	Decomposed count	Atomic %	Atomic Error %
Cd	225674	52.517%	0.096%
Te	203305	47.311%	0.105%
Se	421	0.098%	0.003%

Supplementary Table 1| CdTe Composition Close to the Back Contact: The Cd, Te, and Se composition of a 200 nm tall by 20 nm wide cylindrical region of interest encompassing a region 200 nm - 400 nm from the back contact of a CdTe/CdSe solar cell measured using the peak decomposition tool within IVAS. These results are from a different grain than the one used for

Supplementary Figure 3. The Se content is almost 0 at. %, but there is still less than 0.1 % Se approximately 4 µm from the FTO/$CdTe_xSe_{1-x}$ interface. Se mass spectrum peaks were confirmed in this region and the 0.1% Se measured in this case is above the background signal.

IVAS decomposition analysis			Manual Decomposition used in 1D line profile		
Ion	Decomposed Count	Atomic %	Ion	Decomposed Count	Atomic %
Cd	1171206.121	51.652%	Cd	1555603	50.774%
Se	651009.3252	28.711%	Se	907525	29.621%
Te	438935.9508	19.358%	Te	594617	19.408%
Cl	5982.566692	0.264%	Cl	9808.9166	0.320%
Ga	344.4053059	0.015%	Ga	461	0.015%

Supplementary Table 2| IVAS Peak Decomposition Versus Manual Peak Decomposition. The obtained bulk compositions of an extracted region of the dataset displayed in Figure 5d with Cl enrichment (2 M ions) using the IVAS decomposition analysis and manual decomposition used in the 1D line profiles and proximity histograms. The peak decomposition routine embedded in IVAS 3.6.12 includes a background subtraction routine, while background subtraction was not accounted for in the manual decomposition for the 1D line profiles. Overall, the compositions using both methods are very similar, and the Cl concentration for the 1D line profile is increased by <0.1 at. % due to background. This is minor as the Cl segregation only occurs at the zincblend/wurtzite interface, in which the Cl content increases from 0 at.% to 2 at.% (Figure 5 of the manuscript).
